# Merging van der Waals Materials and Optical Metasurfaces for Cavity Quantum Electrodynamics

**DOI:** 10.1002/nap2.70038

**Published:** 2026-02-23

**Authors:** Luca Sortino, Andreas Tittl, Stefan A. Maier

**Affiliations:** ^1^ Chair in Hybrid Nanosystems, Nanoinstitute Munich, Faculty of Physics Ludwig‐Maximilians‐Universität München Munich Germany; ^2^ School of Physics and Astronomy Monash University Clayton Victoria Australia; ^3^ The Blackett Laboratory Department of Physics Imperial College London London UK

**Keywords:** 2D materials, bound states in the continuum, exciton, metasurfaces, polaritons, van der Waals materials

## Abstract

Flat optical metasurfaces are transforming photonics research by enabling new ways to control light in ultrathin, versatile photonic devices. The rise of quasi‐bound states in the continuum (qBIC) metasurfaces has enabled tailored high‐quality (Q) factor resonances in subwavelength nanostructured thin films, analogous to traditional optical cavities. In this perspective, we explore the emergence of cavity quantum electrodynamics (QED) in optical qBIC metasurfaces, specifically those constructed from van der Waals (vdW) layered materials. Because of their remarkable properties, vdW metasurfaces can support intrinsic optical resonances within the same active material hosting luminescent species, such as excitons or defects, leading to optimal light–matter coupling. This approach of self‐hybridizing the cavity‐emitter system into a single platform effectively overcomes limitations in on‐chip integration of conventional cavities. Combining vdW materials with optically engineered qBIC metasurfaces opens exciting possibilities for exploring nanoscale light–matter interactions. Moreover, the distinctive features of vdW materials, from vertical heterostructures to twist‐angle‐dependent properties, offer a unique platform bridging the condensed matter physics of 2D materials and engineered nanophotonics. We propose that harnessing strong light–matter coupling in vdW‐integrated qBIC metasurfaces will pave the way for next‐generation nanoscale polaritonic devices.

## Graphical abstract

Flat optical metasurfaces supporting quasi‐bound states in the continuum (qBIC) enable cavity‐like, high‐Q resonances in ultrathin nanostructures. This perspective explores the emergence of cavity quantum electrodynamics in qBIC metasurfaces, specifically those made from ultrathin van der Waals nanostructures, where cavities and emitters are seamlessly integrated, enabling efficient light–matter coupling and opening new pathways toward compact, integrated nanoscale polaritonic devices.

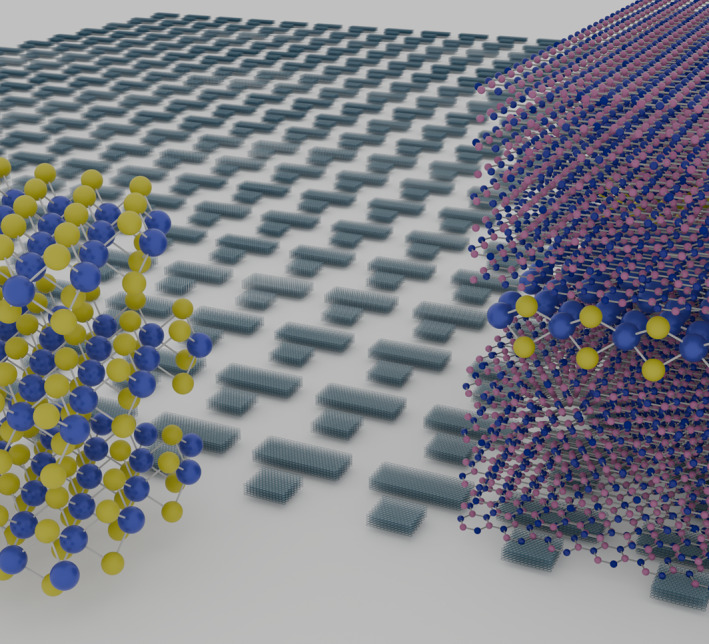

## 1 Introduction

Quantum electrodynamics (QED) investigates the interaction between light (photons) and matter (electrons) and is regarded as one of the most accurate quantum theories developed, having been thoroughly tested in a variety of experiments since its conception [1]. Although the initial ideas emerged from foundational quantum optics, the development of lasers in the 1960s allowed for precise experimental control of photons, leading to breakthroughs in understanding light–matter interactions at the quantum level. Moving beyond the initial atom–photon interaction governed by the fine‐structure constant, when the electromagnetic field is confined within high‐quality optical cavities, its amplitude is enhanced, enabling strong light–matter interaction and giving rise to the field of cavity QED [2]. From the 1980s, research focused on the strong coupling regime, where photons and atoms exchange energy coherently, with the demonstration of vacuum Rabi splitting in atoms and excitons (electron‐hole pairs in semiconductors) in microcavities [3, 4]. The latter led to the discovery of exciton polaritons, hybrid light–matter quasiparticles, and by the 2000s, researchers demonstrated unique properties of exciton‐polariton semiconductor systems, such as nonequilibrium Bose–Einstein condensation [5] and superfluidity [6]. Microcavity exciton polaritons have since become a key focus in cavity QED [7], offering a pathway to novel quantum technologies and devices, from low‐threshold lasing to quantum computation. Although most of the initial studies employed semiconductor quantum wells, with the advent of novel two‐dimensional (2D) materials, such as perovskites and transition metal dichalcogenides (TMDCs), their integration with hybrid nanophotonic structures has provided new perspectives for strong light–matter interactions [8].

**FIGURE 1:**
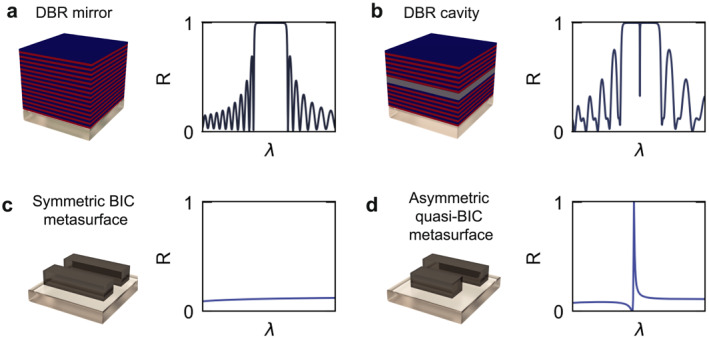
Conceptual similarities between DBRs and qBIC metasurfaces. (a) Illustration of a distributed Bragg reflector (DBR), consisting of alternating layers with different refractive indices and quarter‐wavelength thicknesses. This periodic structure produces a photonic bandgap with near‐unity reflectance, as shown in the reflectance spectrum. (b) DBR‐based optical cavity, where the insertion of a central cavity layer (gray) generates a sharp resonance within the stopband, corresponding to a high‐Q optical mode. (c) Bound state in the continuum (BIC) metasurface with a symmetric double‐rod geometry. Under normal incidence, radiation is suppressed by symmetry protection, and no resonance is observed. (d) Breaking the symmetry by introducing a structural asymmetry between the two rods creates a quasi‐BIC mode, which appears as a sharp high‐Q resonance in the optical spectrum.

Conventional optical cavities can be broadly categorized as either diffraction‐limited or sub‐diffractional. Diffraction‐limited cavities include systems such as photonic crystals and Bragg mirror cavities (Figure 1a,b), where light is periodically reflected by engineering the refractive index contrast between layers. Sub‐diffractional cavities are commonly based on plasmonic or dielectric (Mie) nanophotonic resonators, or nanoantennas, where light can be confined to subwavelength scales, leading to significantly enhanced fields but increasing intrinsic losses.

When arranged in 2D arrays, or metasurfaces, resonant nanostructures can couple coherently and sustain high‐Q resonances through the collective dynamical scattering of individual resonant unit cells. This gives rise to strongly localized near fields that concentrate electromagnetic energy to extreme levels, enabling highly efficient nonlinear processes and challenging conventional approaches [9, 10]. Light–matter interactions with coupled emitters can be enhanced by combining Mie‐type and plasmonic resonator unit cells with array‐based surface lattice resonances, producing substantially higher Q factors realizing the strong light–matter coupling regime with organic molecules and nanomaterials [11]. In these platforms, the optical response is predominantly governed by local geometrical parameters, such as the shape of the individual resonators and the lattice periodicity.

Recently, a novel route for achieving high‐Q resonances in nanophotonic systems has leveraged the physics of bound states in the continuum (BIC) [12, 13]. In these systems, the Q factor, that is, the resonant light confinement, does not depend solely on the geometrical arrangement of the array but rather on the intrinsic symmetry of the unit cell. By carefully breaking this symmetry, a radiative channel is introduced for the intrinsically dark “true” BIC state (Figure 1c), transforming it into a radiative quasi‐BIC (qBIC) resonance (Figure 1d). This process allows for precise tuning of the qBIC features, such as spectral position and linewidth, simply by adjusting the geometry of the unit cell, enabling highly tunable optical responses in ultrathin dielectric metasurfaces.

The strong coupling regime between excitons in 2D materials and optical cavities has been extensively investigated [14, 15]; however, reaching it with optical metasurfaces has so far remained elusive [11, 16], possibly limited by the lack of efficient coupling approaches, which have mainly relied on the transfer of single vdW monolayers on top of prefabricated structures [17]. We propose a new approach to cavity QED using qBIC optical metasurfaces that are monolithically integrated within vdW material systems. Beyond their 2D atomically thin form, layered vdW materials have recently emerged as a promising platform for nanophotonic applications [18], owing to high refractive indexes [19, 20], large anisotropy [21], and the presence of intrinsic excitons and optically active defects, allowing the combination of intrinsic cavity resonances with luminescent emitters, all within the same material [22, 23, 24]. Moreover, the diverse design possibilities and the versatile production of vertical stacks of 2D materials, known as vdW heterostructures, when integrated with the field confinement offered by qBIC metasurfaces, pave the way for precisely engineered multilayered platforms for manipulating light–matter interaction [25].

Although most demonstrations couple emitters to metasurfaces by depositing the active layer on top of a resonant structure [26], vdW materials unlock control of the cavity material itself. Using self‐hybridization in high‐index semiconductors [22] or integrating vdW heterostructures into qBIC metasurfaces [23], one can construct subwavelength cavities directly within the active medium, monolithically unifying cavity and emitter. The resulting freedom to tailor resonance frequency, radiative coupling, and symmetry offers a rich design space for exploring strong light–matter coupling at the nanoscale [18].

In the following sections, we provide a comprehensive perspective on potential research opportunities. First, we introduce the fundamentals of cavity QED and light–matter coupling, then the optical properties of vdW materials for nanophotonics, and finally the control of light–matter interactions in symmetry‐protected qBIC metasurfaces and some examples. The last section presents a perspective on future opportunities in this rapidly evolving field, highlighting the potential of merging the versatile material design of vdW systems with the advanced photonic functionalities of flat optics, opening exciting pathways for the development of next‐generation nanophotonic devices.

## 2 Fundamentals of Cavity QED

Atom–light interactions, described by the Einstein coefficients, were first considered intrinsic to the atomic species itself. The insight that the radiative rate of an electronic transition is affected by its photonic environment, first established in the seminal work of Purcell [27], led to the development of a robust theoretical formalism for cavity QED. However, this theory could not be thoroughly investigated until the development of powerful lasers and its expansion with nanomaterials. Cavity QED is the study of the interaction between light and matter at the quantum level, particularly when the electromagnetic field is confined, or quantized, within an optical cavity. This confinement enhances optical phenomena such as spontaneous emission, absorption, and nonlinear processes, further allowing the observation of coherence and quantum effects otherwise difficult to achieve in free space. Cavity QED systems have since found significant importance in applications such as quantum information processing, quantum communication, and high‐precision sensing [28].

**FIGURE 2:**
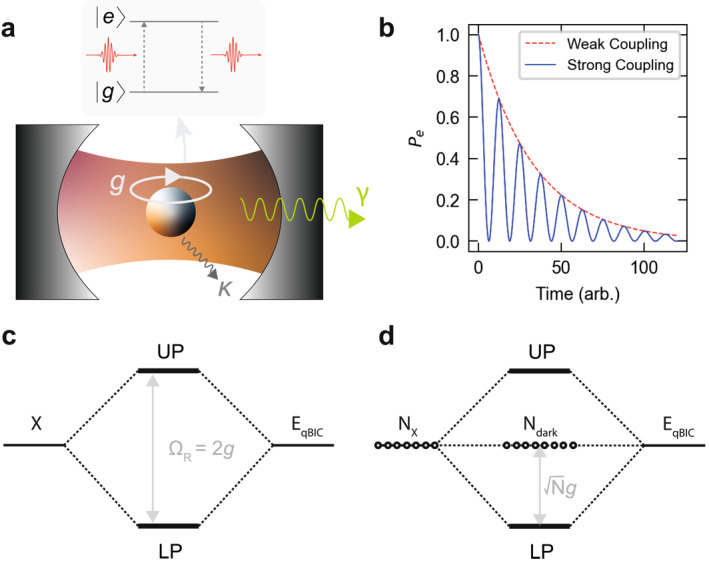
Fundamentals of cavity QED. (a) Illustration of a two‐level system coupled to the photonic mode of an optical cavity. Here, γ denotes the radiative losses, κ denotes the nonradiative losses, and g denotes the coupling strength. (b) Temporal evolution of the excited‐state population $\left(P_{e}\right)$ of the two‐level system in the weak coupling regime (dashed red line), showing exponential decay, in contrast to that in the strong coupling regime (solid blue line), where Rabi oscillations occur between the ground and excited states. (c) Energy diagram of the Jaynes–Cummings model for a single exciton (X) coupled to a qBIC cavity mode ($E_{\mathrm{qBIC}}$). The resulting superposition of light and matter states gives rise to the upper (UP) and lower (LP) polariton branches, with a Rabi splitting energy $\left(\Omega_{R}\right)$ equal to twice the coupling strength (g). (d) Energy diagram of the Tavis–Cummings model for N excitons coupled to a qBIC cavity mode, where a dark exciton population $\left(N_{\mathrm{dark}}\right)$ emerges alongside the strongly coupled system.

The interaction of a two‐level system (e.g., atom, molecule, exciton, or quantum dot) spectrally and spatially coupled with the confined electromagnetic field of an optical cavity (Figure 2a) gives rise to two distinct regimes of light–matter interaction: the weak and strong coupling regimes (Figure 2b). In the weak coupling regime, the system interacts weakly with the electromagnetic field, and thus, the main effect is an enhancement or suppression of the emission rate, the so‐called Purcell effect, depending on the spectral and spatial coupling to the cavity field, directly affecting the local density of states [29]. In the strong coupling regime, instead, the interaction between the system and the cavity photons is strong enough that energy can be coherently exchanged between the two. This leads to the formation of new quantum states, known as dressed states, where the system exhibits Rabi oscillations. The energy is transferred back and forth between the emitter and the photon field in a superposition of the light and matter states, creating new quasiparticles called polaritons.

A fundamental theoretical framework describing the interaction between a two‐level system and a single mode of the cavity field is the Jaynes–Cummings model [30, 31], providing a simple yet powerful description of the quantum nature of light–matter interaction. The Hamiltonian for the Jaynes–Cummings model, in the rotating‐wave approximation, is given by the following equation:

(1) $$H=\hbar \omega_{c}a^{†}a+\frac{1}{2}\hbar \omega_{0}\sigma_{z}+\hbar g\left(\sigma_{+}a+\sigma_{-}a^{†}\right),$$

where $\omega_{c}$ is the frequency of the cavity mode, $\omega_{0}$ is the transition frequency of the two‐level system, $a^{†}$ and a are the photon creation and annihilation operators, respectively, $\sigma_{z}$ is the Pauli matrix that describes the energy levels of the atom, $\sigma_{+}$ and $\sigma_{-}$ are the atomic raising and lowering operators, and g is the coupling strength between the atom and the electromagnetic field mode. The term $\hbar \omega_{c}a^{†}a$ describes the energy of the photons in the cavity, whereas the term $\frac{1}{2}\hbar \omega_{0}\sigma_{z}$ represents the energy of the two‐level system. The exchange of energy between the atom and the field is described by the last term, where $\sigma_{+}a$ represents the absorption of a photon and $\sigma_{-}a^{†}$ the emission process. One of the peculiar phenomena arising from the strong coupling regime is the occurrence of Rabi oscillations (Figure 2b, in blue). In this regime, the two‐level system and the field exchange energy coherently, leading to oscillations in the polariton population characterized by the Rabi frequency. Furthermore, when in resonance with the cavity mode, the energy levels of the combined emitter‐cavity system split into two distinct levels, known as vacuum Rabi splitting (Figure 2c). This energy splitting, equal to 2g, is a direct consequence of the coherent exchange of energy, and the new eigenstates (polaritons) represent the quantum superposition of light and matter states.

A generalization of the Jaynes–Cummings model to a system with multiple two‐level systems interacting with a single electromagnetic mode, more descriptive of practical solid‐state systems, is the Tavis–Cummings model [32]. The Hamiltonian for the Tavis–Cummings model is given by the following equation:

(2) $$H=\hbar \omega_{c}a^{†}a+\sum_{j=1}^{N}\frac{1}{2}\hbar \omega_{0}\sigma_{z}^{\left(j\right)}+\hbar g\sum_{j=1}^{N}\left(\sigma_{+}^{\left(j\right)}a+\sigma_{-}^{\left(j\right)}a^{†}\right),$$

where N is the number of emitters in the cavity, $\sigma_{z}^{\left(j\right)}$ is the Pauli matrix for the jth emitter, $\sigma_{+}^{\left(j\right)}$ and $\sigma_{-}^{\left(j\right)}$ are the raising and lowering operators for the jth emitter. When identical emitters are coupled to the cavity, they behave as a single effective system with an enhanced Rabi splitting compared with that in the single‐atom case, scaled by the effective coupling strength $\sqrt{N}g$ (Figure 2d). An addition to the Tavis–Cummings model is indeed the description of collective states where the emitters interact cooperatively with the cavity field, such as superradiance, where photons are emitted collectively, or the generation of quantum‐entangled states.

The strong coupling regime can reach even substantial fractions of the optical resonance energy, entering the so‐called ultrastrong coupling regime [33, 34]. In this limit, the interaction energy becomes comparable to the bare emitter or cavity resonance, leading to a breakdown of the conventional rotating‐wave approximation. This regime opens unexplored opportunities for controlling vacuum field fluctuations, accessing nonperturbative QED, and engineering novel photonic and material functionalities beyond the standard strong coupling framework.

## 3 Nanophotonics Based on van der Waals Materials

Nanophotonic devices based on high‐refractive‐index materials have established themselves as an efficient alternative to traditional metal‐based resonators. By avoiding intrinsic plasmonic losses and combining electric and magnetic Mie resonances, dielectric materials have provided a reliable means to control optical responses at the nanoscale [35, 36]. The introduction of common high‐index dielectrics such as silicon, germanium, and gallium phosphide has expanded the landscape for shaping light at subwavelength scales, ultimately leading to the flourishing research on dielectric nanoresonators and metasurfaces [37, 38].

**FIGURE 3:**
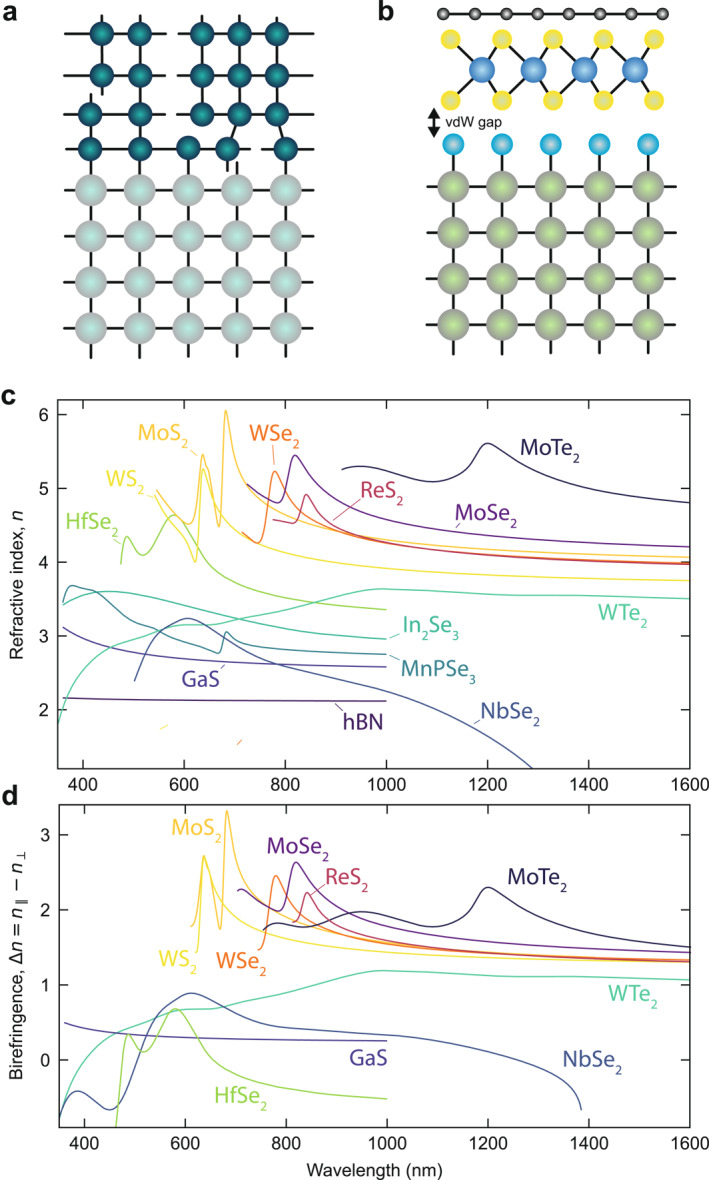
Optical properties of van der Waals materials. (a,b) Illustration of a semiconductor heterostructure interface between conventional covalent materials (a) and vdW materials (b). In covalent heterostructures, lattice mismatch between adjacent layers creates defects and imperfections, degrading crystalline quality and limiting device performance. In contrast, in a heterostructure based on 2D layers, a van der Waals gap separates each layer from its neighbors and from the substrate, preserving interface quality and allowing greater freedom in material choice. (c,d) Refractive index (c) and birefringence (d) (as the difference between in‐ and out‐of‐plane components) of representative vdW materials. Data are adapted from Refs. [19, 20].

However, conventional dielectric materials suffer from lattice mismatch when different materials are bonded together (Figure 3a), posing fabrication challenges and restricting material combinations. This limits the optoelectronic quality with the introduction of defects and constrains the design of advanced metasurfaces based on multilayered optical systems. In contrast, the development of layered vdW materials has introduced new flexibility in designing vertical heterostructures, owing to the absence of covalent bonds between layers (Figure 3b). The term “vdW material” broadly refers to a class of layered crystals with strong in‐plane bonding and weak vdW forces in the out‐of‐plane direction. They can be exfoliated down to single atomic sheets, as pioneered by the discovery of graphene 20 years ago [39]. Although these materials have gained attention across various fields, their role in nanophotonics has focused predominantly on harnessing the monolayer form via functionalization of passive structures [40]. Several fields of research have been advancing the understanding of light–matter interactions in 2D materials, from graphene plasmonics [41, 42] to strong coupling in 2D TMDC semiconductors [43] and appealing nonlinear optical responses [44, 45]. Additionally, vdW materials offer a variety of crystalline symmetries, expanding the design possibilities for photonic devices [46].

Among 2D semiconductors, the class of TMDCs has been extensively studied, owing to robust excitons at room temperature and their nonlinear optical properties, together with their impact on scaling down electronic components [47]. For photonic applications, monolayer TMDCs exhibit unique capabilities, including feasible integration, 2D polaritons [48], and native single‐photon sources [49]. Furthermore, ultrathin optical devices based on monolayer TMDCs, such as metalenses [50] and waveguides [51], have been realized, highlighting their promising applications as building blocks of advanced nanophotonic and optoelectronic technologies.

In recent years, the optical properties of thin multilayer films of vdW materials, ranging from tens to hundreds of nanometers in thickness, have garnered significant interest for nanostructured and integrated photonics [18, 52, 53, 54]. This attention stems from their high refractive indices (Figure 3c), attributed to strongly polarizable atoms and their layered structures [55], which facilitate intrinsic optical resonances and efficient light confinement. Furthermore, the wide range of available vdW materials offers broad spectral transparency windows, which are ideal for low‐loss applications, along with large optical anisotropy (Figure 3d) combined with high nonlinear efficiencies, significantly expanding the design potential for advanced nanophotonic applications and toward the miniaturization of optical components [56].

Initial demonstrations in vdW‐based nanophotonics have highlighted the potential of high‐index vdW materials for waveguide miniaturization [57] and for overcoming limitations in current integrated photonics [58]. The high refractive index in these materials enables the formation of geometric optical resonances in nanostructures. Nanophotonic structures based on TMDCs have been demonstrated, including nanodisks supporting anapole states [59, 60], lasing in whispering gallery mode nanodisks [61], dimer structures [62], and atomically precise metamaterials [63]. Additionally, functional devices such as optical modulators [64] and nonlinear optical metasurfaces [65] have been developed. The combination of high refractive indices and strong nonlinear efficiencies makes vdW‐based nanophotonics an appealing approach for enhancing nonlinear optical processes [66]. In this context, studies on bulk 3R‐phase TMDCs have demonstrated significant second‐harmonic‐generation enhancements [67, 68], comparable to those observed in standard nonlinear crystals, leading to the demonstration of entangled photon‐pair sources [69, 70] and recent integration into qBIC metasurfaces [71, 72].

The rapid evolution of vdW nanophotonics is tightly linked to advances in the growth and assembly of large‐area devices. Early demonstrations based on chemical vapor deposition (CVD) growth [73] have already shown the potential to fabricate multilayered nanophotonic structures from bulk ${\text{MoS}}_{2}$ thin films, enabling scalability beyond exfoliation‐based approaches. The development of automated and deterministic assembly techniques is expected to further enhance scalability and yield [74], paving the way toward complex vdW heterostructures and large‐scale nanophotonic systems and expanding the capabilities and broad applicability of next‐generation optical devices.

## 4 Light–Matter Interaction in qBIC Metasurfaces

BICs are resonant states that exist within the continuum of radiation modes yet remain confined through destructive interference, preventing their coupling to the environment [13, 75, 76, 77, 78]. In photonics, BICs can be made accessible to the far field by introducing symmetry‐breaking perturbations in their geometrical configuration [12]. In dielectric metasurfaces, such symmetry breaking gives rise to quasi‐BIC (qBIC) resonances, which are characterized by extremely high‐Q factors and Fano‐like spectral line shapes. Owing to the strong light–matter interaction enabled by qBIC resonances, diverse phenomena including highly efficient nonlinear processes [79], directional upconversion [80], and quantum state generation [81] have been demonstrated.

**FIGURE 4:**
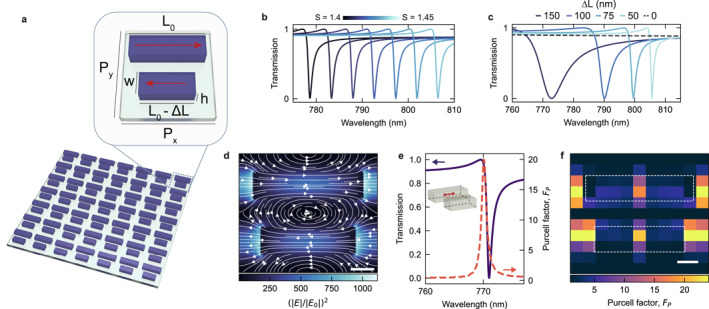
Coupling dipolar emitters to qBIC metasurfaces. (a) Illustration of the double‐rod asymmetric qBIC metasurface. Inset: unit cell geometry with main design parameters. Red arrows indicate the dipole moments at the qBIC resonance. (b) Numerical simulations showing spectral tuning of the qBIC resonance by applying a scaling factor S to the in‐plane unit cell parameters (excluding height). (c) Numerical simulations of linewidth tuning of the qBIC resonance as the asymmetry parameter ΔL is increased. (d) Numerical simulations of the in‐plane electric field intensity calculated at the qBIC resonance frequency. (e) Simulated optical transmission of a qBIC metasurface (blue line) together with the spectral dependence of the Purcell factor $\left(F_{P}\right)$, maximized in resonance with the qBIC transmission dip. Inset: position of the in‐plane dipole, aligned with the qBIC near field for maximal coupling. (f) Spatial map of the Purcell factor for an in‐plane dipole aligned along the *x*‐axis, evaluated at the qBIC resonance frequency for different dipole positions in the plane at half the nanorod height. All panels are adapted from Ref. [24].

For simplicity, here we focus on qBIC metasurfaces with a design based on a unit cell with a double‐rod asymmetric geometry (Figure 4a), although the following principles apply to many other geometrically asymmetric qBIC designs [82]. The choice of this structure lies in the dominance of the optical response by the longitudinal dipole resonance of the rod structure (red arrow in Figure 4a), which provides a single, spectrally isolated qBIC resonance. In a symmetric structure, the dipole moments of each Mie resonant nanorod produce two counter‐oscillating dipoles oriented along the longitudinal direction of the rods. If these are perfectly symmetric, they cancel each other out, resulting in a dark BIC state. By reducing one of the two rods via the parameter ΔL, the symmetry breaking induces an asymmetry in the vectorial sum of the two dipole moments when driven by a resonant light field (Figure 4d). The asymmetry introduces an effective radiative coupling channel, acting as an optical cavity mode and manifesting as a dip in the transmission spectrum with a distinctive Fano profile.

Beyond acting as subwavelength high‐Q factor optical cavities, qBIC metasurfaces offer exceptional freedom for maximizing emitter‐cavity coupling, allowing to tune both the linewidth and the spectral position of the resonance. By applying a scaling factor, S, which multiplies the initial in‐plane parameters of the unit cell design (except for the height), it is possible to finely tune the qBIC spectral position (Figure 4b), a process that is limited solely by the material and layer thickness. On the other hand, the asymmetry parameters allow to finely tune the spectral linewidth of the qBIC resonance (Figure 4c) and thus the cavity Q factor, allowing to control the losses and maximize the field enhancement for critical coupling [22]. This tunability of high‐Q factor resonances in subwavelength films is an unprecedented opportunity to develop novel approaches for cavity QED, where nanoscale electromagnetic fields and structured materials combine in a single system.

The chosen double‐rod geometry provides an efficient platform for light–matter coupling specifically with in‐plane dipole emitters, commonly found in 2D semiconductors. The near field generated at the qBIC resonance (Figure 4d) is composed mostly of in‐plane electric components and aligns with the longitudinal axis of the nanorod. Nevertheless, the multipolar nature of photonic BIC [83] allows to also design out‐of‐plane resonant fields by mixing higher‐order multipolar modes [84], which would maximize coupling with out‐of‐plane emitters.

To integrate luminescent emitters with metasurfaces, the transfer or growth of the active material on top of passive nanostructures is especially detrimental to research on 2D materials, as the transfer of thin layers usually yields defects and fractures, leading to nonreproducible and complex responses. On the other hand, by placing the dipole inside the nanoresonator itself (Figure 4e), the strong coupling to the qBIC near field leads to a large enhancement of the spontaneous emission rate, via the Purcell effect, by maximizing the local density of states in resonance with the qBIC mode frequency. Figure 4f shows the spatial mapping of the Purcell factor values for an in‐plane dipole within the plane of a qBIC metasurface unit cell. The maximal values for embedded dipoles are observed at the center of each nanorod, as expected from the maximum value of the near‐field simulations.

## 5 Examples of qBIC vdW Metasurfaces

### 5.1 Self‐Hybridized Exciton Polaritons in TMDC Metasurfaces

**FIGURE 5:**
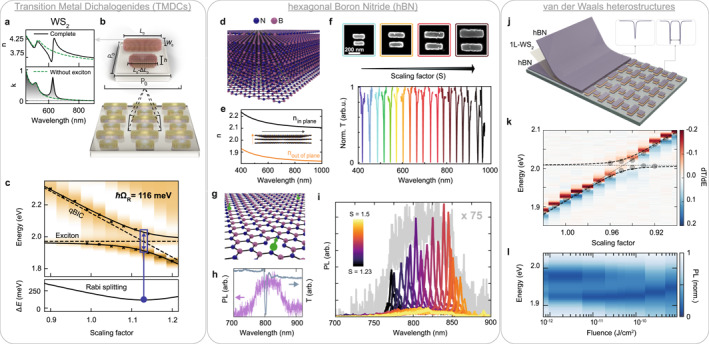
Combining qBIC metasurfaces and vdW materials for enhanced light–matter coupling. (a) Real and imaginary parts of the refractive index of bulk ${\text{WS}}_{2}$. (b) Illustration of a double‐rod asymmetric qBIC metasurface fabricated from thin ${\text{WS}}_{2}$ films (40–100‐nm thick), with main unit cell design parameters. (c) Experimental transmission spectra of a set of ${\text{WS}}_{2}$ qBIC metasurfaces, showing the anticrossing between the ${\text{WS}}_{2}$ exciton and the qBIC resonance, the hallmark of strong light–matter coupling, with a Rabi splitting of 116 meV at room temperature. (d) Illustration of a multilayer crystal of hBN. (e) Top: electron micrographs of hBN metasurface unit cells with different scaling factors. Bottom: experimental qBIC transmittance spectra across the visible range for the corresponding metasurfaces. (f) Atomistic structure of a native boron vacancy ($V_{B}^{-}$) defect in hBN. (g) Photoluminescence (PL) emission of $V_{B}^{-}$ defects (purple) and transmission spectrum (gray) of a high‐Q metasurface resonance. (h) PL spectra of $V_{B}^{-}$ defects coupled to hBN metasurfaces with different scaling factors, showing spectral matching between defect emission (in gray) and the metasurface resonance. (i) Illustration of a vdW heterostructure metasurface consisting of a monolayer (1 L) ${\text{WS}}_{2}$ encapsulated in two thin hBN layers and patterned to sustain qBIC resonances for strong coupling with 2D excitons. (j) Experimental transmittance spectra of vdW heterostructure metasurfaces as a function of the scaling factor, exhibiting a Rabi splitting of ∼30 meV at room temperature. (k) Power‐dependent PL emission from a vdW heterostructure metasurface, showing saturation from the strong to weak coupling regime. Panels a–c are adapted from Ref. [22], d–f from Ref. [86], g–h from Ref. [24], and j–l from Ref. [23].

In TMDCs, and particularly in monolayers, strong excitonic resonances persist up to room temperature owing to their large binding energies, which can exceed several hundred meV. The coupling of 2D TMDCs with optical cavities has been extensively studied over the last decade, facilitated by the favorable integration of atomically thin sheets into conventional photonic architectures [85]. An alternative approach to achieving strong exciton‐photon coupling exploits the intrinsically high refractive index of multilayer TMDCs in combination with resonant nanophotonics. In nanostructured layers, carefully engineered geometries support photonic resonances enabled by the large TMDC refractive index, which can couple to the intrinsic excitons of the material (Figure 5a). To fabricate the samples, TMDC layers are mechanically exfoliated onto the substrate, rather than grown epitaxially, and subsequently patterned by top‐down nanofabrication, leaving the active nanostructures directly on the substrate. The first demonstration of this concept was realized in TMDC nanodisks sustaining anapole resonances [59]. However, such systems are strongly limited by the geometric constraints of the anapole mode and by their low Q factors. In contrast, the versatile control and high‐Q resonances of qBIC metasurfaces enable strong exciton–photon interactions over a much broader parameter space.

The strong light–matter coupling regime has been demonstrated in monolithic qBIC metasurfaces fabricated from ${\text{WS}}_{2}$ multilayers thinner than 50 nm (Figure 5b) [22]. Figure 5c shows the reflectance of a set of ${\text{WS}}_{2}$ qBIC metasurfaces with increasing scaling factors, shifting the qBIC resonance across the ${\text{WS}}_{2}$ exciton energy near 2 eV. A clear anticrossing between the two resonances confirms the strong light–matter coupling regime, with Rabi splitting values exceeding 100 meV under ambient conditions. Moreover, by carefully tailoring the cavity linewidth via the asymmetry parameters, the balance of the qBIC losses can be finely controlled for maximization of the emitter‐cavity coupling strength.

Exciton polaritons in self‐hybridized systems have also been reported in one‐dimensional ${\text{WS}}_{2}$ gratings [73, 87], further demonstrating the versatility of TMDC nanophotonics. In addition, the deliberate use of out‐of‐plane symmetry breaking in metasurfaces has enabled the realization of chiral qBIC resonances that, when integrated with ${\text{WS}}_{2}$ multilayers, have shown chiral‐selective strong coupling [88]. The adoption of multilayers, however, inevitably sacrifices the appealing physics inherent to 2D monolayers, narrowing the design space. This limitation highlights the need for alternative strategies that preserve monolayer characteristics while still enabling versatile device architectures.

### 5.2 Visible Resonances With hBN Metasurfaces

The second 2D material to be isolated after graphene was hexagonal boron nitride (hBN), which shares a similar crystal structure (Figure 5d) but is an insulator with a wide bandgap of ∼6 eV. It has become widely used in 2D material research, particularly as a substrate for electronic devices and as an encapsulation layer that improves the quality of 2D heterostructures [89]. In nanophotonics, hBN exhibits several exciting properties: chemical robustness, a refractive index n>2 with transparency across the visible spectrum (Figure 5e), and the ability to support phonon polaritons at infrared frequencies [90]. Notably, similar to diamond, hBN can also host a variety of luminescent defects, making it a promising candidate for quantum optical applications [91].

The relatively low refractive index of hBN makes it difficult to realize Mie‐type optical resonances in the visible range, necessitating the use of suspended photonic crystal membranes [92]. In contrast, symmetry‐protected qBIC states in metasurfaces leverage structural asymmetries of the unit cell rather than the absolute refractive index, enabling versatile designs for visible resonances in hBN. Recently, qBIC metasurfaces fabricated from exfoliated hBN crystals have achieved Q factors above 200 across the visible spectrum (Figure 5f), enabled by the favorable transparency window of hBN [86]. These values are currently limited by fabrication, and through optimized processing and the implementation of inverse design techniques, Q factors exceeding 1000 have been demonstrated [93].

The high‐Q factors of hBN metasurfaces have enabled effective coupling with optically addressable spin defects native to hBN [94], thereby opening new pathways for quantum technologies [24] (Figure 5g). In these experiments, metasurfaces are fabricated to resonate with the spectral emission of the defects (Figure 5h), whereas boron vacancies are introduced directly into the hBN structures by ion implantation. Although the defects couple only weakly to the enhanced electromagnetic field of the qBIC resonance, the interaction nonetheless yields an order‐of‐magnitude enhancement in photoluminescence (Figure 5i), accompanied by a reduction of the spectral linewidth to below 4 nm, for enhancing efficiency and resolution in quantum sensing and imaging applications. This demonstration establishes the basis for an ultrathin cavity system constructed entirely from hBN, in which luminescent emitters such as 2D excitons or optically active defects can be integrated within the same platform.

Expanding the versatility of qBIC metasurfaces, recent research has demonstrated chiral hBN metasurfaces capable of engineering polarization‐sensitive responses [93, 95], as well as infrared qBIC designs that extend functionality beyond the visible range [96]. In parallel, monolithic hBN structures such as waveguides have shown promise for coupling to luminescent defects [97], providing routes to enhanced single‐photon emission and potential on‐chip photon routing. Together, these advances position hBN‐based metasurfaces and integrated structures as a uniquely versatile platform for next‐generation quantum nanophotonics.

### 5.3 van der Waals Heterostructure Metasurfaces

The advent of multilayered vdW heterostructures has reshaped fundamental approaches to nanodevice fabrication by enabling the mechanical stacking of diverse 2D crystals into arbitrary architectures [98]. This atomic‐scale control over the assembly of multilayered structures offers unique potential to tailor the functionalities of optoelectronic devices. In nanophotonics, multilayered structures are essential for engineering precise control of light, from metamaterial concepts to Bragg reflectors. However, many of these concepts are challenging to implement when thicknesses become fractions of a single wavelength, and vdW heterostructures may provide a novel route to overcome these limitations or to explore entirely new regimes. In particular, encapsulation with hBN is essential for producing high‐quality vdW samples, as it preserves and enhances the intrinsic properties of embedded 2D layers, showing linewidth‐limited exciton emission from TMDC monolayers [99]. In this direction, initial work has demonstrated hBN‐based heterostructures for generation and waveguiding of excitonic light [100, 101] and light–matter coupling in Fourier grating metasurfaces [102].

Merging the concept of hBN qBIC metasurfaces with encapsulated 2D material layers could further extend the strong coupling regime in ultrathin heterostructures. For example, a single ${\text{WS}}_{2}$ monolayer encapsulated between two 60‐nm hBN films can be patterned into a resonant metasurface [23], resulting in isolated submicron heterostructure pillars (Figure 5j). Figure 5g shows the transmittance of a set of heterostructured metasurfaces, exhibiting an anticrossing between the ${\text{WS}}_{2}$ monolayer exciton near 2 eV and the qBIC resonance, with a room‐temperature Rabi splitting of 30 meV. Beyond confirming strong coupling, the metasurface‐coupled exciton polaritons also exhibit pronounced nonlinearities, including saturation of strong coupling and signatures of lasing (Figure 5g), with thresholds orders of magnitude lower than those in conventional approaches [23]. These results establish hBN‐based metasurfaces as a high‐Q cavity, adding an additional layer via vdW heterostructure engineering and providing a platform for enhanced light–matter interactions for low‐power nanoscale devices.

## 6 Perspectives and Outlook

The convergence of high‐Q metasurfaces and vdW materials has opened new avenues for controlling light–matter interactions at the nanoscale, expanding the toolbox of metaoptics, quantum photonics, and polaritonics [11, 40]. Although significant progress has been made, the field remains at a formative stage, with several outstanding challenges and promising research directions, which will shape its evolution.

A central challenge is the realization of ultrahigh‐Q resonances in compact, planar metasurface geometries that are compatible with the integration of atomically thin quantum materials. Although qBICs offer a powerful mechanism to achieve high Q factors, they are sensitive to structural asymmetries and fabrication imperfections [103]. Furthermore, vdW materials often introduce additional optical losses due to absorption and scattering, which can degrade the efficiency of modulators or coherence in cavity‐coupled systems. Moreover, transfer techniques for vdW materials can introduce mechanical and structural defects, limiting overall device performance.

Another major step lies in the precise spatial and spectral alignment between confined photonic modes and quantum emitters or excitons within vdW materials. The open and delocalized nature of metasurface resonances, although advantageous for integration, complicates deterministic positioning and efficient coupling of single emitters with nanoscale precision [24]. In addition, limited dynamic tunability, particularly postfabrication, restricts the ability to actively control resonance conditions or switch between coupling regimes. Finally, from a scalability perspective, the reproducible integration of metasurfaces with diverse vdW materials remains technically demanding [85]. Variability in material and interface quality or alignment precision can hinder the development of reliable, large‐scale photonic platforms for practical applications.

Overcoming these challenges will require coordinated advances in design, materials, and fabrication. Emerging approaches such as topological metasurfaces offer enhanced robustness against disorder [104], potentially enabling high‐Q operation under less stringent fabrication tolerances. On the material side, encapsulation techniques, environmental stabilization, and surface passivation will be critical for preserving the coherence properties of vdW excitons and minimizing nonradiative losses. The development of ultraclean interfaces and deterministic stacking methods will further enhance device reproducibility and performance [105]. In parallel, inverse design and machine learning algorithms promise to accelerate the discovery of metasurface geometries optimized for strong coupling, with tailored mode profiles, field enhancements, and emission directionality [106].

As the field matures, we anticipate that metasurface‐based cavity QED systems will evolve from proof‐of‐concept demonstrations to scalable technologies that merge the precision of nanophotonics with the physics of quantum materials. Several frontiers are emerging that highlight both the scientific richness and the potential for transformative applications. In the following sections, we outline some potential areas of interest.

### 6.1 Heterostructure Engineering

The flexibility in stacking and interface engineering in vdW materials can be harnessed to control material composition, crystalline orientation, and interlayer spacing, creating a unique experimental playground for realizing metasurface concepts and physical phenomena otherwise inaccessible in traditional platforms. Moreover, the concept of vdW heterostructure metasurfaces discussed before can be extended to the broad library of 2D materials and their heterostructures, expanding the scope of vdW‐based devices. By leveraging the unique electronic and optical properties of these systems, multifunctional platforms can be envisioned in which material engineering and photonic control are seamlessly integrated. The incorporation of metaoptics into 2D architectures thus constitutes a logical step forward, enabling reconfigurable, ultrathin devices that combine quantum functionality, optical tunability, and scalable integration for future nanophotonic and quantum technologies.

Additionally, recent advances in integrating microelectromechanical systems (MEMS) for on‐chip modulation of interlayer spacing [107, 108] and twist‐angle control [70] now provide dynamic and reversible tuning of interlayer interactions, unlocking emergent properties in bilayer and moiré systems that bridge optics, topology, and correlated condensed matter physics [109]. The optical landscape is further enriched by crystalline interface engineering, which enables deliberate symmetry breaking, enhances nonlinear generation [110], and supports the design of advanced nonlinear [111] and chiral [112] layered photonic structures. Extending these paradigms to moiré photonics offers new opportunities to manipulate excitons and polaritons in patterned quantum landscapes [113].

### 6.2 Quantum Nanophotonics

Beyond delocalized excitons, vdW materials host native quantum emitters, including single‐photon sources in hBN and TMDCs [114]. Coupling these emitters to high‐Q resonances would enhance their emission efficiency for scalable on‐chip quantum optics and open pathways toward coherent light–matter interactions at the single‐photon level. Initial work in this direction has been realized by coupling hBN to passive BIC metasurfaces [115], whereas integration of monolayer TMDC emitters is still lacking. Although precise spatial control of emitter placement remains challenging, emerging site‐controlled approaches such as ion implantation [116] may enable deterministic positioning. Additionally, efficient spontaneous parametric down conversion has been reported in vdW crystals, and their integration with self‐hybridized metasurfaces could provide new routes for engineering both the efficiency and the emission characteristics [117].

Although such advances would represent a major step forward in the realization of compact, integrated quantum light sources, metasurfaces are still predominantly implemented in free‐space configurations, limiting their direct applicability to fully integrated photonic circuits. Nevertheless, waveguide‐integrated metasurfaces have already been demonstrated for controlling light coupling and propagation [118], and qBIC metasurfaces could operate as high‐Q optical cavities, analogous to photonic crystal or Fabry–Pérot resonators, for which efficient on‐chip integration has been successfully achieved [119]. Both qBIC metasurfaces and vdW materials provide powerful platforms for proof‐of‐concept demonstrations of novel quantum optical phenomena, particularly those arising from the intrinsic properties and exceptional external tunability of 2D materials. The complementary role in exploring new regimes of light–matter interaction, such as strong light–matter coupling and active control of quantum emission, may open new directions not easily accessible in conventional semiconductor systems.

### 6.3 Metasurface‐Engineered Polaritons

Exciton polaritons coupled to optical metasurfaces provide a powerful platform for investigating polariton interactions and condensation. Recent studies on vdW semiconductor metasurfaces have demonstrated large nonlinearities at low polariton densities [23] and room‐temperature condensation via the efficient feeding of the condensate via indirect excitons [120]. When combined with the capability of metasurfaces to engineer photonic landscapes, these effects enable enhanced control over polariton states and their dynamics by tailoring mode dispersion, confinement, and radiative coupling at the nanoscale. This level of control allows the design of interaction strengths, relaxation pathways, and condensate configurations, opening access to quantum phenomena such as strongly nonlinear polariton fluids, engineered condensation landscapes, and nonequilibrium quantum phases in tailored polaritonic systems.

### 6.4 Chiral and Anisotropic Polaritons

Chiral light–matter interactions are a rapidly emerging direction in optical metasurfaces [121]. With vdW materials, it is possible to design meta‐atoms that break in‐plane or out‐of‐plane symmetries through twist, stacking, or anisotropy, which could hold promise to generate valley‐selective and spin‐polarized states. By merging these options with excitonic strong coupling [88], these phenomena hold promise for chiral quantum optics platforms, where photons can be routed, filtered, or emitted with well‐defined handedness.

In parallel, many vdW materials naturally exhibit strong optical anisotropy, making them ideal candidates for polarization control at the nanoscale. The deliberate combination of structural asymmetry with intrinsic anisotropy enables richer design opportunities, potentially giving rise to exotic polaritonic states associated with exceptional points [122] and topological properties [123]. These non‐Hermitian singularities not only enhance sensitivity but also open the door to unconventional light manipulation, such as asymmetric transmission and nonreciprocal polariton transport [104, 124]. The convergence of chirality, anisotropy, and strong coupling in vdW metasurfaces thus points toward a new class of reconfigurable nanophotonic platforms, where symmetry breaking becomes a tool for engineering robust and highly controllable quantum states of light.

### 6.5 Magneto‐Polaritons

The discovery of intrinsic magnetic order in van der Waals materials such as ${\text{CrI}}_{3}$ and FeGeTe has established 2D magnets as a rapidly growing field of research, with profound implications for spintronics and optoelectronics [125]. When stabilized by hBN encapsulation, overcoming poor ambient stability, these materials could unlock strong magneto‐optical interactions. Additionally, antiferromagnetic compounds such as CrSBr [126] and NiPS3 [127] are environmentally stable and offer additional opportunities for exciton‐magnetism coupling and nonlinear magneto‐optics. CrSBr, in particular, has recently shown highly tunable excitonic resonances under small magnetic fields, together with an exceptionally high refractive index (n∼30), making it a compelling candidate for strongly confined photonic resonances [128] and for realizing self‐hybridized magneto‐exciton polaritons [129]. Initial studies have also reported tunability of qBIC resonances with magnetic fields [130], opening intriguing prospects for controllable light–matter interactions at the intersection of magnetism and nanophotonics.

### 6.6 Tunable Devices

An appealing feature of vdW materials, particularly in their monolayer form, is their exceptional tunability enabled by a wide range of control parameters, including electric fields, mechanical strain, ion intercalation, dielectric environment engineering, layer stacking, and twist angle [113, 131, 132]. The integration of 2D materials with tunable metasurfaces represents a compelling direction for further advances in active nanophotonic platforms. Initial examples of active components based on single layers have been demonstrated for tunable metalenses [50] and spatial modulator gratings [133, 134]. When combined with the excitonic and nonlinear properties of vdW materials, such platforms offer the ability to modulate polariton resonances, tailor emission spectra, and control light–matter interactions. This synergy holds particular promise for ultrafast modulators and adaptive optical components, where vdW heterostructures serve not only as the active medium but also as an enabling material platform for scalable, reconfigurable metasurfaces. Unlike conventional passive architectures, tunable optical metasurfaces can dynamically reconfigure phase and amplitude in real time [135], and, particularly, qBIC metasurfaces provide novel approaches to tune optical resonances via all‐optical modulation of permittivity and refractive index [136, 137]. To achieve high speed and scalability, electro‐optic control has remained the preferred route [138]. Current electro‐optic modulation strategies in metasurfaces frequently rely on liquid crystals [139], phase‐change materials [140], or MEMS actuation [107]. These, however, limit speed and stability, as such integration of electro‐optic materials with high Pockels coefficients, such as LiNbO [141] and organic molecules [142], offers higher modulation speeds. For vdW‐based metasurfaces, a central challenge is the integration of efficient carrier injection and electrical control. So far, active nonlinear ${\text{WS}}_{2}$ vdW metasurfaces have been demonstrated via thermal tuning [143]. Achieving reliable electrical injection at the level of individual meta‐atoms remains an open challenge, and most architectures rely on 1D metagrating to accommodate electrode designs for electrical modulation [135]. Ongoing advances in vdW heterostructure engineering, where atomically thin semiconductors can be combined with metallic contacts, tunnel barriers, or ferroelectric layers, offer promising routes to overcome this limitation and provide a foundation for electrically pumped polaritonic devices, tunable nonlinear metasurfaces, and emission control.

## 7 Conclusions

In conclusion, the integration of layered van der Waals materials with qBIC‐based optical metasurfaces offers a versatile and reconfigurable platform for next‐generation nanophotonics. By uniting advanced material engineering with high‐performance metaoptics, this approach enables unprecedented opportunities ranging from novel cavity QED schemes and exploration of 2D excitons to the integration of native single‐photon sources and scalable on‐chip quantum devices, establishing vdW metasurfaces as a transformative interface between condensed matter physics and photonics. Looking ahead, harnessing stacking and interface engineering, moiré physics, chiral and magnetic effects, and scalable electrical control will be crucial for unlocking the full potential of vdW nanophotonics, paving the way for practical, reconfigurable, and multifunctional quantum and nonlinear optical technologies at the atomic scale.

## Author Contributions

**Luca Sortino:** conceptualization, visualization, writing – original draft, writing – review and editing. **Andreas Tittl:** conceptualization, writing – review and editing. **Stefan A. Maier:** conceptualization, writing – review and editing.

## Funding

Funded by the European Union (ERC, METANEXT, 101078018 and EIC, NEHO, 101046329). Views and opinions expressed are however those of the author(s) only and do not necessarily reflect those of the European Union, the European Research Council Executive Agency, or the European Innovation Council and SMEs Executive Agency (EISMEA). Neither the European Union nor the granting authority can be held responsible for them. This work was also funded by the Deutsche Forschungsgemeinschaft (DFG, German Research Foundation) under Germany’s Excellence Strategy (EXC 2089/1 ‐ 390776260), Sachbeihilfe MA 4699/7‐1 and the Emmy Noether program (TI 1063/1); the Bavarian program Solar Energies Go Hybrid (SolTech) and the Center for NanoScience (CeNS). L.S. acknowledges funding support through a Humboldt Research Fellowship from the Alexander von Humboldt Foundation. S.A.M. additionally acknowledges the Lee‐Lucas Chair in Physics and the Australian Research Council.

## Data Availability

Data sharing is not applicable to this article as no datasets were generated or analyzed during this study.
